# An Evaluation of a Syndromic Molecular Panel in Optimising the Microbiological Diagnosis and Antimicrobial Therapy of Suspected Osteoarticular Infections in Paediatric Patients

**DOI:** 10.3390/diagnostics15050566

**Published:** 2025-02-26

**Authors:** Marilena Agosta, Venere Cortazzo, Manuela Onori, Barbara Lucignano, Gianluca Vrenna, Martina Rossitto, Maria del Carmen Pereyra Boza, Valeria Fox, Marco Roversi, Antonio Musolino, Andrzej Krzysztofiak, Laura Lancella, Marco Giordano, Francesco Falciglia, Ottavia Porzio, Alberto Villani, Carlo Federico Perno, Paola Bernaschi

**Affiliations:** 1 Microbiology and Diagnostic Immunology Unit, Bambino Gesù Children’s Hospital, IRCCS, 00165 Rome, Italy; marilena.agosta@opbg.net (M.A.); manuela.onori@opbg.net (M.O.); barbara.lucignano@opbg.net (B.L.); mdcarmen.pereyraboza@opbg.net (M.d.C.P.B.); carlofederico.perno@opbg.net (C.F.P.); paola.bernaschi@opbg.net (P.B.); 2 Multimodal Laboratory Medicine, Bambino Gesù Children’s Hospital, IRCCS, 00165 Rome, Italy; gianluca.vrenna@opbg.net (G.V.); martina.rossitto@opbg.net (M.R.); valeria.fox@opbg.net (V.F.); 3 PhD Program in Immunology, Molecular Medicine and Applied Biotechnology, University of Rome Tor Vergata, 00133 Rome, Italy; marco.roversi@opbg.net; 4 Residency School of Pediatrics, University of Rome Tor Vergata, 00133 Rome, Italy; antonio.musolino@opbg.net; 5 Infectious Diseases and Immunoinfectivology Unit, Bambino Gesù Children’s Hospital, IRCCS, 00165 Rome, Italy; andrzej.krzysztofiak@opbg.net (A.K.); laura.lancella@opbg.net (L.L.); 6 Orthopedics and Traumatology Unit, Bambino Gesù Children’s Hospital, IRCSS, 00165 Rome, Italy; marco.giordano@opbg.net (M.G.); francesco.falciglia@opbg.net (F.F.); 7 Clinical Laboratory Unit, Bambino Gesù Children’s Hospital, IRCCS, 00165 Rome, Italy; ottavia.porzio@opbg.net; 8 General Pediatric and Infectious Disease Unit, Pediatric Emergency Medicine, Bambino Gesù Children’s Hospital, IRCCS, 00165 Rome, Italy; alberto.villani@opbg.net

**Keywords:** paediatric osteoarticular infections, septic arthritis, rapid diagnostic, molecular syndromic approach

## Abstract

**Background/Objectives:** Paediatric osteoarticular infections (POAIs) present unique diagnostic and therapeutic challenges. Microbiological culture (MC) is typically time-consuming and lacks sensitivity, especially when patients have received antibiotics. The BIOFIRE^®^ Joint Infection Panel (BJIP) is a syndromic molecular assay for the direct identification of most pathogens causing POAIs. **Methods:** We evaluated BJIP in 17 synovial fluids, and then, we retrospectively assessed its utility in 93 off-label specimens (i.e., 25 purulent fluids/biopsies and 68 whole blood samples). All specimens were collected from October 2022 to March 2024 from paediatric patients admitted at the Bambino Gesù Children’s Hospital in Rome. **Results:** A bacterial pathogen was isolated in only one of 17 synovial fluid cultures, while BJIP identified eight additional microorganisms in MC-negative cases. The most frequently detected pathogen was *S. aureus* (44.5%, 4/9). BJIP performance in synovial fluids showed an overall positive percentage agreement (PPA) and negative percentage agreement (NPA) of 100% and 88.1%, respectively, compared to MC. All positive results (n/N = 9/17) were considered medically significant, with an increase in NPA to 100%. In purulent fluids/biopsies, BJIP and MC were concordant in 72% of cases (n/N = 18/25), with a per-sample PPA and NPA of 90% and 60%, respectively. For whole blood samples, almost all samples were negative by both methods (i.e., reference blood culture and BJIP), and the molecular test did not enable any further microbiological diagnosis. **Conclusions:** The BIOFIRE^®^ Joint Infection Panel rapidly and accurately enabled or excluded a diagnosis of a POAI (~1 vs. 24–96 h for MC), optimising antimicrobial therapy.

## 1. Introduction

Paediatric osteoarticular infections (POAIs), i.e., osteomyelitis and septic arthritis, are top paediatric emergencies with an estimated incidence of about 8 cases per 100,000 children and 4 to 10 cases per 100,000 children per year, respectively [1]. Misdiagnosis and delayed treatment may increase the likelihood of disseminated complications with serious and permanent sequelae [2,3]. Paediatric osteoarticular infections are most commonly caused by bacterial pathogens, with *Staphylococcus aureus* remaining the primary causative agent, responsible for about 70–90% of cases across all age groups [4]. Other notable pathogens include *Streptococcus pyogenes*, frequently isolated in younger children; Group B Streptococci and *Escherichia coli* should be considered in neonates and immunocompromised patients. *Kingella kingae* is increasingly recognised as an important cause of osteoarticular infections in children under 5 years, and its prevalence has been highlighted by the use of Polymerase Chain Reaction (PCR)-based methods [5,6]. Indeed, according to the European Society for Paediatric Infectious Diseases (ESPID) Bone and Joint Infection Guidelines (ESPID Guidelines), the diagnosis and management of bone infections rely on a combination of clinical, imaging, and microbiological criteria, including the use of molecular diagnostic methods [4]. Although enhanced culture-based techniques such as the implant sonication and inoculation of synovial fluid into blood culture bottles has improved sensitivity, there remains a high percentage of culture-negative cases, ranging from 5 to 42% [7,8], especially in patient under antibiotic treatment at the time of the cultural test [7,8,9]. In these circumstances, the culture-negative rate can further increase to 64% [9,10]. The use of a molecular assay ensures a timely diagnosis and the prompt administration of targeted therapy. The BIOFIRE^®^ Joint Infection Panel (BJIP) is a multiplex PCR-based test that simultaneously detects 39 targets (31 pathogens and 8 resistance genes) in less than 1 h [11]. The inclusion of molecular diagnostic tools such as BJIP may improve pathogen detection, particularly in pre-treated patients where culture methods show limited sensitivity.

Here, we evaluated the performance and the clinical impact of BJIP in optimising the diagnosis and antibiotic treatment of POAIs. Although, the BJIP is only approved by the FDA to test synovial fluid [12], we retrospectively assessed the potential utility of BJIP in diagnosing POAIs, even in samples other than synovial fluid [13], i.e., purulent fluids/biopsies and whole blood samples collected from paediatric patients with the suspicion of osteoarticular infection.

## 2. Materials and Methods

### 2.1. Study Design and Participants

In 2022, the Bambino Gesù Children’s Hospital (Rome, Italy) adopted the BIOFIRE^®^ Joint Infection Panel (BJIP) as a routine diagnostic tool for assessment of suspected osteoarticular infections or septic arthritis in association with traditional microbiological culture (MC). This retrospective single-centre observational–descriptive study included consecutive patients aged 0–21 years old with a diagnostic MC and/or a BJIP test requested simultaneously at the time of hospital admission or during their hospitalisation from 25 October 2022 to 31 March 2024. Patient demographic and clinical information were collected from electronic medical records.

### 2.2. Clinical Diagnostic Criteria to Define POAIs

According to national guidelines and internal protocols, clinical suspicion of osteomyelitis or septic arthritis in paediatric patients was based primarily on a combination of clinical presentation, laboratory findings, and imaging studies. Clinically, children may present with an acute onset of joint pain, swelling, erythema, and limited range of motion, often accompanied by systemic symptoms such as fever. Laboratory evaluations typically reveal elevated inflammatory markers, including C-reactive protein (CRP) and erythrocyte sedimentation rate (ESR). Leukocytosis may also be observed, although its absence does not exclude the diagnosis. Imaging modalities play a crucial role in diagnosis; while plain radiographs are often the initial step, they may appear normal in early stages. Then, magnetic resonance imaging is considered the gold standard due to its high sensitivity in detecting early osteomyelitis and assessing adjacent soft tissue involvement. Additionally, Kocher criteria can aid in differentiating septic arthritis from transient synovitis in children presenting with acute hip pain. These criteria include no weight bearing on the affected side, fever > 38.5 °C, ESR > 40 mm/h, and a white blood cell count > 12,000 cells/µL. The probability of septic arthritis increases with the number of criteria present [4]. Syndromic testing and/or microbiological culture requests as well as antimicrobial treatments were at the discretion of the treating physicians based on the aforementioned criteria for clinical suspicion of POAIs.

### 2.3. Microbiological Investigation for Synovial Fluid Specimens

To ensure comprehensive microbiological assessment, synovial fluid samples were immediately processed for both microbiological conventional culture and BJIP testing. Synovial fluid samples were collected from children in some cases of suspected osteomyelitis to exclude concomitant osteoarthritis or septic arthritis, given the anatomical proximity of bones and joints and the overlapping of clinical features. Joint fluid collection also represented a less invasive diagnostic approach, especially in paediatric patients.

Clinical specimens were routinely analysed according to the standardised protocols of the microbiology laboratory. Briefly, synovial fluids (if there was enough sample volume) were inoculated into blood culture vials (BCV) in accordance with hospital practices and international recommendations for paediatric populations [4,14]. Blood culture vials were incubated at 37 °C in a Bactec 9240/70FX BC system (BD Diagnostics, Franklin Lakes, NJ, USA) for at least five days unless they previously tested positive [4,14,15,16]. Blood culture vials tested positive were sub-cultured on aerobic and anaerobic solid media, and agar plates were incubated at 35–37 °C overnight in aerobic and anaerobic conditions, as described below.

Otherwise, in case of low sample volume, synovial fluid samples were directly cultured on solid blood agar, chocolate agar, and Sabouraud agar for the detection of aerobic bacteria and yeasts and on Schaedler anaerobic agar (all from bioMérieux, Marcy-l’Étoile, France) for cultivation of anaerobic bacteria. Plates were incubated at 35–37 °C in 5% CO2 for 3 days and in an anaerobic atmosphere for 5 days. All colonies grown on agar plates were identified using Matrix-Assisted Laser Desorption Ionisation-Time of Flight Mass Spectrometry (MALDI-TOF MS; Bruker Daltonics, Bremen, Germany). Strains were tested for their antimicrobial susceptibility by broth microdilution method using the MicroScan Walkaway system (Beckman Coulter Inc., Carlsbad, CA, USA) according to clinical breakpoints based on the European Committee on Antimicrobial Susceptibility Testing (EUCAST) tables (version 14.0) [17].

### 2.4. Off-Label Specimens

According to clinical suspicion of joint infection and/or the positivity obtained by traditional microbiological culture, we collected other residual biological specimens different from synovial fluids (i.e., purulent fluids/biopsies and whole blood samples) to assess the diagnostic performance of the BJIP and to evaluate the hypothetical impact on reducing time for diagnosis. Residual biological specimens were stored in cryovials at −80 °C until further analysis.

Specimens included were purulent fluids drained close to the painful limb as well as biopsies taken in proximity to the compromised limb/bone. Whole blood samples were also processed to investigate hematogenous osteomyelitis and in case the collection of the aforementioned biological samples was difficult to obtain. These samples were analysed independently and were not necessarily matched with the synovial fluid samples collected from the same patients.

Off-label specimens were processed routinely for microbiological conventional diagnosis by means of culture on solid media for purulent fluids/biopsies and blood culture for whole blood.

#### 2.4.1. Microbiological Investigation for Purulent Fluids/Biopsies

Purulent fluids were directly cultured on solid media while biopsy specimens were sonicated prior to culture to facilitate microbial growth. Briefly, biopsies were vortexed in a container with sterile saline for 30 s and then sonicated in an ultrasonic bath sonicator for 1 min at maximum intensity (BactoSonic; BANDELIN electronic GmbH & Co. KG, Berlin, Germany). An amount of 100 µL of sonicated solution was sub-cultured on agar plates. Solid media and incubation conditions as those described for synovial fluid were used.

#### 2.4.2. Microbiological Investigation for Whole Blood Samples

Whole blood samples were processed by BJIP, and results were compared to reference blood culture. Blood cultures were inoculated in accordance with hospital practices and international recommendations for paediatric populations [4,14]. Briefly, each BCV was inoculated with 1–3 mL (Bactec Peds Plus/F medium) or 8–10 mL (Bactec Plus Aerobic/F medium, Bactec Lytic/10 Anaerobic/F medium vials) of whole blood and incubated at 37 °C in a Bactec 9240/70FX BCV system (BD Diagnostics, Franklin Lakes, NJ, USA). Blood culture vials that did not yield a pathogen were incubated for at least 5 days. Blood culture vials that tested positive were sub-cultured on aerobic and anaerobic solid media and incubated as previously described.

### 2.5. BIOFIRE^®^ Joint Infection Panel

An amount of 200 µL of fresh clinical synovial fluid sample or 200 µL of residual biological specimens retrospectively collected (i.e., purulent fluids/biopsies and whole blood samples) were processed with BIOFIRE^®^ Joint Infection Panel (BJIP) (bioMérieux, Marcy-l’Etoile, France) for pathogen and resistance gene identification according to the manufacturer’s instructions. BIOFIRE^®^ Joint Infection Panel enabled multiplex detection of 39 molecular targets, and it was based on two nested 16s rRNA amplifications for each of these. The targets included 29 Gram-negative/Gram-positive bacteria frequently causing joint infections, 2 Candida species, and 8 antimicrobial resistance genes (Table 1).

### 2.6. Statistical and Data Analysis

Continuous (expressed as median values with interquartile ranges [IQR]) or categorical (expressed as numbers with percentages) variables were analysed using the Mann–Whitney U test or Fisher’s exact test, respectively. The BJIP result was considered true negative (TN) when both the BJIP and microbiological culture were negative. The BJIP result was considered a true positive (TP) when both BJIP and microbiological culture were positive with the same microorganisms. In case of discrepancy between the BJIP result and microbiological culture, the BJIP result was classified as false negative (FN) or false positive (FP) based on the result of the culture. Agreement between the two methods was assessed by calculating the positive percentage agreement (PPA), that is, ‘sensitivity’ (PPA = 100 × [TP/{TP + FN}]), and the negative percentage agreement (NPA), that is, ‘specificity’ (NPA = 100 × [TN/{TN + FP}]). Moreover, the “adjusted” PPA and the “adjusted” NPA were calculated, considering as true positive the discrepant results with positive BJIP/negative microbiological culture, based on the clinical suspicion of POAIs. The 95% CIs for sensitivity and specificity were calculated using the exact Clopper Pearson method. The analyses were conducted using SPSS v.23 and R 4.0.5.

## 3. Results

### 3.1. Study Population and Samples

The study recruited all consecutive patients aged 0–21 years old admitted to Bambino Gesù Children’s Hospital in Rome (Italy) for suspected osteoarticular infections because of acute pain/trauma/bone fractures from October 2022 to March 2024.

**Figure 1:**
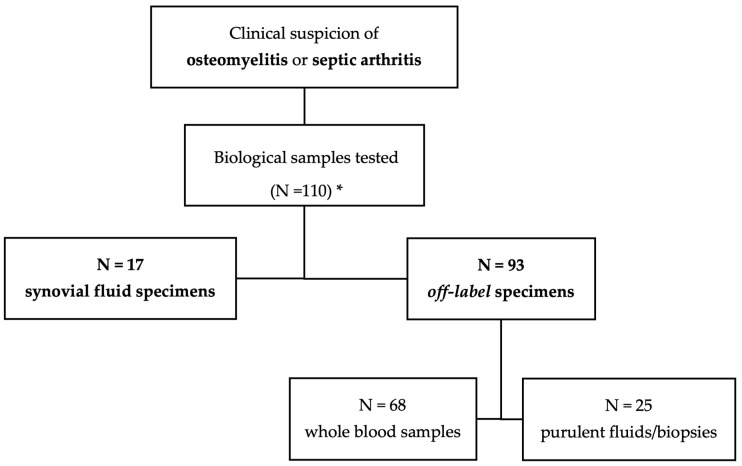
Overall tested specimens by BIOFIRE^®^ Joint Infection Panel (BJIP). * Samples collected from 96 paediatric patients.

The final study population consisted of 96 patients with a total of 110 biological samples tested using the BJIP: 17 synovial fluid specimens were collected from 17 patients, and 93 off-label specimens (i.e., 25 purulent fluids/biopsies and 68 whole blood samples) were collected from 79 patients (Figure 1).

Table 2 reports the characteristics of the study population and related joint infections. The median (interquartile range, IQR) age was 9.8 (5.3–13.6) years. Middle age groups (2–18 years) accounted for 80.2% of the overall population (n/N = 77/96), with 17 suspected POAIs investigated in toddlers, 35 in children, and 25 in adolescents. Among 96 patients with clinical suspicion of POAIs, 60.4% were males, and 39.6% were females.

Clinical suspicion of septic arthritis was slightly higher than that of osteomyelitis (49.0% vs. 44.8%, respectively). White blood cell count and procalcitonin were mostly within normal limits, respectively, in 89.8% and in 83% of patients with available data, while C-reactive protein and erythrocyte sedimentation rate were increased, respectively, in 71.3% and 66.7% of patients with available information. The mainly involved or compromised joint/bones were hips (n/N = 32/96, 33.3%), followed by knees (n/N = 22/96, 23.0%) and ankles (n/N = 11/96, 11.4%).

Overall, clinical suspicion of infection was confirmed by microbiological culture and/or a positive BJIP result in only 27% of cases (n/N = 26/96). Of these 26 confirmed cases, in 92.3%, the infection was monomicrobial, while in 7.7%, the infection was polymicrobial; moreover, in 11.5% of cases, the infection was also caused by anaerobic (n/N = 3/26) and antibiotic-resistant bacteria (n/N = 3/26).

### 3.2. Performance of BIOFIRE^®^ Joint Infection Panel on Synovial Fluid Specimens

Table 3 shows overall findings for synovial fluid samples (N = 17) analysed in the study, i.e., diagnosis and involved joint, as well as microbiological results obtained from culture (MC) and BIOFIRE^®^ Joint Infection Panel (BJIP).

According to national guidelines and imaging finding, in all cases, the diagnosis confirmed the clinical suspicion with 3 cases of osteomyelitis and 14 of septic arthritis, although the causative microorganism were not always detected.

Cases with low cellularity in synovial fluid were also carefully reviewed for alternative diagnoses, including transient synovitis of the hip. Differential diagnosis was at the discretion of the treating physician, and this condition was not found.

Results of MC and BJIP were concordant in 53% of cases (n/N = 9/17): in particular, results were both negative in 47% (n/N = 8/17) of cases, while in only one case, MC confirmed the presence of S. pyogenes, also detected by BJIP. In the remaining cases, BJIP resulted positive, but no microorganism was isolated in culture. The positive results, obtained by MC and/or BJIP, were considered significant since in all cases (n/N = 9/17), they reflected clinical suspicion. The combined performance of BJIP and MC in the diagnosis of POAIs from synovial fluid specimens is reported in Table 4. The most frequently isolated/detected pathogen was S. aureus (44.5%, 4/9), followed by S. pyogenes and K. kingae (22.2% each, 2/9) and H. influenzae (11.1%, 1/9).

The per-organism PPA was 100% (95% CI: 2.5–100) for S. pyogenes. The per-organism NPA ranged from 76.5% (95% CI: 50.1–93.2) for S. aureus to 94.1% (95% CI: 71.3–99.9) for H. influenzae. The “adjusted” PPA and the “adjusted” NPA (calculated as described above) increased to 100% (95% CI: 66.4–100 and 95% CI: 93.4–100, respectively) for all detected species by BJIP.

**Figure 2:**
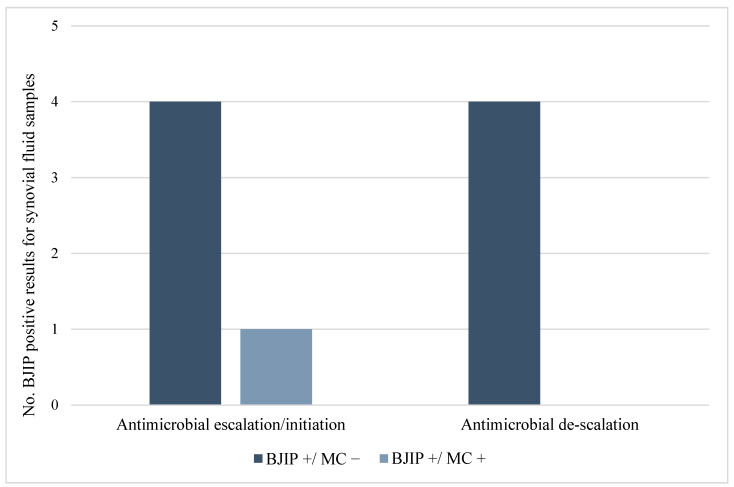
Antimicrobial therapy interventions performed according to BJIP-positive results for synovial fluid samples from paediatric patients.

The eight microbial species detected only by BJIP were all found in synovial fluids from patients who had already empirically received a potentially active antimicrobial treatment for at least 48 h at the time of testing. Examining medical records, we found that based upon BJIP results, antibiotic therapy was changed in 100% (n/N = 9/9) of cases: in particular, the antibiotic was escalated/initiated in 55.6% (n/N = 5/9), and it was de-escalated in 44.4% (n/N = 4/9) (Figure 2) of cases.

### 3.3. Performance of BIOFIRE^®^ Joint Infection Panel on Off-Label Specimens

Table 5 reports the combined performance of BJIP and MC for the 93 off-label specimens, consisting of 25 purulent fluids/biopsies and 68 whole blood samples. For purulent fluids/biopsies, results of MC and BJIP were concordant in 72% of cases (n/N = 18/25): in particular, results were either concordant negative or concordant positive in the same percentage of 36% (n/N = 9/25). BIOFIRE^®^ Joint Infection Panel resulted positive but no microorganism was isolated in culture in 24% of cases (n/N = 6/25); conversely, in only one case, BJIP resulted negative while a microorganism was isolated from concomitant MC. For the 68 whole blood samples, MC and BJIP results were concordant in 100% of cases.

The per-sample PPA was 90% (95% CI: 55.5–99.8) and 100.0% (95% CI: 2.5–100), while the per-sample NPA was 60.0% (95% CI: 32.3–83.7) and 100.0% (95% CI: 94.6–100) for purulent fluids/biopsies and whole blood, respectively. As for synovial fluids, the “adjusted” PPA and the “adjusted” NPA were calculated for off-label specimens according to the same interpretative criteria. The two values remain the same for whole blood samples, while the PPA and the NPA increased to 93.8% (95% CI: 69.8–99.8) and to 100% (95% CI: 66.4–100), respectively, for purulent fluids/biopsies.

Overall, the most frequently isolated/detected pathogen was S. aureus (70.6%, 12/17); in 2/12, *mec*A/C and MREJ genes were detected by BJIP, while in 1/12, methicillin-resistant S. aureus (MRSA) was isolated from femur biopsy culture, but neither the microorganism nor the antimicrobial resistance gene were detected by BJIP. In three cases, the BJIP diagnosed infections caused by anaerobic bacteria from one biopsy and from two purulent fluids, respectively. In the first case, the infection was monomicrobial, and it was caused by Bacteroides fragilis; in purulent fluids, infections were polymicrobial with Parvimonas micra and Peptoniphilus spp. detected in one case and Anaerococcus prevotii/vaginalis, S. aureus, and S. lugdunensis in the other.

The BJIP identification results (~1 h) were available in a time significantly shorter than that necessary for the growth of the microorganism in culture (mean ~56 h) or that required for blood culture to turn positive and subsequent final microorganism identification (mean [SD] = 10.9 [5.5] hours; *p* < 0.001 and mean [SD] = 65.7 [24.5] hours; *p* < 0.001, respectively) [18,19].

## 4. Discussion

In recent years, the emergence of molecular techniques has offered a promising solution to expedite pathogen identification and promptly guide targeted antimicrobial treatment in the diagnosis of joint infection. In fact, there remain many culture-negative cases, especially if a patient is already receiving antibiotics [7,8,9,10].

In this study, we primarily evaluated the performance of the BIOFIRE^®^ Joint Infection Panel (BJIP) test directly on synovial fluid specimens collected from paediatric patients with suspected osteoarticular infections. Our results confirmed that the use of the panel increased diagnostic capacity in comparison with traditional culture techniques for synovial fluids, especially for microorganisms that are difficult to culture with a high sensitivity/positive percent agreement and specificity/negative percent agreement. A more in-depth analysis of the synovial fluid sample results, as reported in Table 3, highlighted the diversity of identified pathogens. The presence of bacteria, such as *S. aureus*, *S. pyogenes*, *K. kingae*, and *H. influenzae*, points out the variety of paediatric osteoarticular infections (POAIs). Of note, according to clinical suspicion, BJIP enabled the microbiological diagnosis of eight additional cases of joint infections (resulted negative by traditional culture), thus increasing the microbiological recovery by 47%, in accordance with what was previously described by Wouthuyzen-Bakker [9]. In children of all ages, *S. aureus* was the most frequently detected pathogen involved in joint infections, as reported in other studies [3,4], followed by *S. pyogenes* and *K. kingae*, which are the two other most common bacteria-causing POAIs. *K. kingae*, in particular, is the most prevalent microorganism in septic arthritis in children < 5 years old [4]. Our results perfectly reflect the above statements, especially the detection of *K. kingae* in two synovial fluids collected from two female patients aged 1 and 2 years old, respectively, both with a diagnosis of knee septic arthritis. The identification of this microorganism is crucial since the infection could present with a slightly different clinical picture when compared to *S. aureus* and other microorganisms, inducing a symptomatology that mimics respiratory viral infections [20]. For this reason, *K. kingae* incidence in paediatric arthritis may be underestimated, but the use of molecular methods has significantly improved its detection [21,22,23,24].

Intriguingly, BJIP diagnosed a case of osteomyelitis by *H. influenzae* in a 48-day-old infant. *H. influenzae*, especially type b (Hib), was reported as a common cause of native septic arthritis in young children before the introduction of the Hib-conjugated vaccine [25,26]. Since the introduction of routine vaccination against *H. influenzae* and *S. pneumoniae* as well, these bacteria have no longer been detected in osteoarticular infections in patients under five years old [27]. In our case, considering the infant’s age, he had not yet received the first dose of the anti-Hib vaccine since the first vaccination dose is expected to be administered two months after birth (starting from the 61st day of life) according to the Italian Ministry of Health [28]. Although the BJIP test does not distinguish between encapsulated *H. influenzae* and non-typeable *H. influenzae* strains, this case underlines the importance of molecular testing in unvaccinated infants, where invasive infections by *H. influenzae* may still occur. Based on these results’ discrepancy investigation and as reported in other studies [11], the BJIP-positive/microbiological-culture-negative cases are likely true positives since 100% of these cases were correctly related to the clinical suspicion. The reduction of diagnostic time was a significant advantage in using the molecular panel, especially in samples where traditional microbiological culture yielded negative results. This enabled the early initiation of targeted antimicrobial therapy in 100% of cases with a positive result on synovial fluids, thus improving patients’ clinical outcome.

The promising results obtained on synovial fluids with BJIP prompted us to evaluate whether BJIP could be helpful in defining the diagnosis of osteoarticular infection by also testing non-synovial fluid specimens. BIOFIRE^®^ Joint Infection Panel successfully identified pathogens in six additional cases using purulent fluids and biopsies, nearly doubling the microbiological confirmation rate compared to culture alone. To do so, BJIP and traditional MC were performed on 93 off-label samples (i.e., 25 purulent fluids/biopsies and 68 whole blood samples) retrospectively collected from paediatric patients with suspected POAIs. Noteworthy, our findings highlight a good panel performance in such samples, demonstrating the potential role of BJIP in expanding its diagnostic applications, enabling a more comprehensive and timely diagnosis of POAIs. The use of various molecular syndromic panels on off-label specimens has already been described in the literature, both to diagnose unresolved cases of infection or to evaluate the performance of a syndromic panel on a large number of samples [29,30]. Despite the FDA only approving the BJIP for synovial fluid samples [7], our results show a good agreement between the traditional microbiological culture and the molecular panel performed on the aforementioned off-label samples, with per-sample PPAs of 90% and 100.0% and per-sample NPAs of 60.0% and 100.0% for purulent fluids/biopsies and whole blood, respectively, indicating its effectiveness in diagnosing POAIs. As described for synovial fluids, BJIP would have allowed the microbiological diagnosis of six more cases of suspected POAIs in addition to the nine cases confirmed by culture, almost doubling the possibility of diagnosis. The high number of these positive cases found in this sample group is probably due to the bias of the selected patients, as BJIP was performed only in those with high clinical suspicion. Interestingly, among the six positive purulent fluids/biopsies specimens by BJIP and not by culture, we found three infections caused by anaerobic bacteria. The difficulty of diagnosing polymicrobial infections or culturing anaerobic microorganisms is well described in the literature [24,31]. One reason could be the overgrowth of one of the strains that hide the growth of more slowly growing organisms, but in our case no microorganisms had been detected in culture, and it is more likely that the previous antimicrobial treatment made growth in culture impossible. Then, we cannot assume that these results were false positive, as they confirmed the clinical suspicion. Conversely, the BJIP failed to detect methicillin-resistant *S. aureus* (MRSA) in a femur bone biopsy specimen. As far as we reconstructed from the medical record, the BJIP was performed on the same sample previously processed for microbiological culture. We can hypothesise that since it was a bone biopsy, the steps to which the sample was subjected before culturing on solid media (i.e., sonication and enrichment in broth culture) have facilitated and made possible the growth of the microorganism in culture. Instead, the use of the mere liquid obtained from the sonication of the bone fragment to perform the molecular test did not prove suitable for detecting the microorganism. This could explain the negative result of the BJIP compared to culture. This discrepancy emphasises the importance of integrating BJIP results with traditional culture methods, particularly in cases where pre-processing steps, such as sonication, enhance culture-based recovery, taking into account that this procedure may introduce contamination, which should be considered when interpreting results. For whole blood samples, we found that almost all samples were negative by both methods (i.e., reference blood culture and BJIP) and that the molecular test did not enable any further microbiological diagnosis. These findings suggest that BJIP has limited diagnostic yield when applied to whole blood in POAI cases. According to current guidelines [4], blood culture with appropriate volumes should always be performed, but in our experience, the use of whole blood samples for the diagnosis of POAIs by BJIP perhaps should not be encouraged. Indeed, in seven patients for whom we tested both synovial fluids or residual purulent fluids/biopsies and whole blood specimen, the molecular panel detected a microorganism in the former but not in whole blood samples. In these patients, blood cultures were also negative, suggesting that the infection was purely localised and that taking a sample directly from the site of infection increases the probability of diagnosing the infection itself [22].

Considering our results, the most relevant aspect with the use of BJIP on the off-label samples is that the result would have been available to clinicians in a time significantly shorter than that necessary for the growth of the microorganism in culture (~1 vs. 56 h) or that required for blood culture to turn positive and then subsequent final microorganism identification (mean [SD] = 10.9 [5.5] hours; *p* < 0.001 and mean [SD] = 65.7 [24.5] hours; *p* < 0.001, respectively, as reported by Lucignano B. and colleagues), thus enabling the rapid initiation or escalation/de-escalation of a previously administered antimicrobial therapy [18,19]. Therefore, these patients would have benefitted too from a faster identification result. In fact, from a medical record analysis of these retrospective collected off-label samples, all patients with positive microbiological culture underwent a change in antimicrobial therapy but only after the time necessary for microorganism growth in culture.

Intriguingly, we did not observe growth by coagulase-negative staphylococci (CONS) or *Cutibacterium* spp. in culture for any of the studied specimens. Actually, these are common pathogens in chronic and implant-associated osteoarticular infections [11]. In the paediatric setting this seems not to be a “problem”, and the panel is suitable for the diagnostic needs of these patients [4,12,32,33].

The retrospective and monocentric nature of our study accounted for some limitations. In fact, the samples analysed, both synovial fluids and off-label specimens, were selected and collected exclusively based on the clinical suspicion of POAIs, which could also be incorrect or hypothetical, especially if no causative pathogen was identified. Therefore, a key limitation of our study is the retrospective design, which may introduce selection bias.

Furthermore, future prospective studies are necessary to validate these findings in larger, well-defined cohorts with age-stratified diagnostic approaches, as the prevalence of pathogens varies significantly among neonates, infants, and adolescents.

Another limitation is that discordant results (i.e., BJIP positive and microbiological culture negative) were not further investigated with complementary molecular methods to assess the diagnosis, and the BJIP-positive result was considered significant solely based on physician’s clinical suspicion. Moreover, the low sensitivity of culture (5.88% for synovial fluids) would require a thorough analysis of factors influencing bacterial recovery, including preanalytical sample handling, previous antibiotic exposure, and culture conditions.

Although the performance of BJIP in synovial fluids of paediatric patients has been evaluated in other studies [11,20,32], to the best of our knowledge, the present study is the first in which the use of BJIP has been described in diagnosing POAIs, even in samples different from synovial fluids exclusively in a paediatric setting. However, further prospective research is needed to evaluate this potential use even in different paediatric clinical contexts.

In conclusion, it is important to underline that BJIP cannot (at least at this point) replace traditional microbiological culture, which remains essential for definitive diagnosis and especially for antimicrobial susceptibility testing. Our results provide concrete evidence of the effectiveness of integrating BJIP into clinical practice for an accurate diagnosis and optimal management of POAIs. The syndromic panel allowed for faster identification of pathogens (both in the case of difficult-to-cultivate microorganisms and for patients already receiving empiric antibiotic therapy) with increased diagnostic sensitivity, rapid optimal change in antimicrobial therapy, and reduced diagnostic turnaround time.

## Figures and Tables

**Table 1:** Pathogens and antibiotic resistance genes detected by the BIOFIRE^®^ Joint Infection Panel.

Gram-Negative Bacteria	Gram-Positive Bacteria	Yeasts	Antimicrobial Resistance Genes
**Aerobic**	**Aerobic**		
*Citrobacter* spp.	*Enterococcus faecalis*	*Candida* spp.	IMP
*Enterobacter cloacae* complex	*Enterococcus faecium*	*Candida albicans*	KPC
*Escherichia coli*	*Staphylococcus aureus*		NDM
*Klebsiella aerogenes*	*Staphylococcus lugdunensis*		OXA-48-like
*Klebsiella pneumoniae* group	*Streptococcus* spp.		VIM
*Morganella morganii*	*Streptococcus agalactiae*		
*Proteus* spp.	*Streptococcus pyogenes*		CTX-M
*Pseudomonas aeruginosa*			
*Salmonella* spp.			*van*A/B
*Serratia marcescens*			
**Fastidious**	**Fastidious**		*mec*A/C and MREJ (MRSA)
*Haemophilus influenzae*	*Streptococcus pneumoniae*		
*Kingella kingae*			
*Neisseria gonorrhoeae*			
**Anaerobic**	**Anaerobic**		
*Bacteroides fragilis*	*Anaerococcus prevotii/vaginalis*		
	*Clostridium perfringens*		
	*Cutibacterium avidum/granulosum*		
	*Finegoldia magna*		
	*Parvimonas micra*		
	*Peptoniphilus*		
	*Peptostreptococcus anaerobius*		

**Table 2:** Baseline characteristics of the study population and related joint infections.

Characteristics	Description	Overall Paediatric Patient N = 96
Paediatric age groups, n (%)	Neonate (1 week to 1 month)	1 (1.0)
	Infant (1 month to 1 year)	13 (13.6)
	Toddler and preschool (2–5 years)	17 (17.7)
	School age child (6–12 years)	35 (36.5)
	Adolescent (13–18 years)	25 (26.0)
	Young adult (19–21 years)	5 (5.2)
Sex, n (%)	Male	58 (60.4)
	Female	38 (39.6)
Clinical suspicion, n (%)	Osteomyelitis (OM)	43 (44.8)
	Septic arthritis (SA)	47 (49.0)
	Both (OM + SA)	6 (6.2)
Involved joint/bone, n (%)	Hip	32 (33.3)
	Knee	22 (23.0)
	Ankle	11 (11.4)
	Others (vertebral column, jaw, tibia, wrist, shoulder, humerus)	31 (32.3)
White blood cell count, n (%) ^a^ [median (IQR)]	Within normal limits	97 (89.8) [8.29 (6.52–10.79)]
	Neutrophilic leukocytosis	11 (10.2) [17.46 (16.31–18.31)]
C-reactive protein (mg/dL), n (%) ^b^ [median (IQR)]	≥0.5	77 (71.3) [5.62 (2.77–10.32)]
	<0.5	31 (28.7) [0.06 (0.04–0.18)]
Procalcitonin (ng/mL), n (%) ^c^ [median (IQR)]	≥0.5	13 (16.7) [0.63 (0.52–1.88)]
	<0.5	65 (83.3) [0.08 (0.05–0.17)]
Erythrocyte sedimentation rate (mm/h), n (%) ^d^ [median (IQR)]	≥15	46 (66.7) [31.5 (23.0–46.75)]
	<15	23 (33.3) [5.0 (4.0–9.0)]
Clinical suspicion of infection, n (%)	with microbiological confirmation	26 (27.0)
	without microbiological confirmation	70 (73.0)
Joint infection, n (%)	Monomicrobial	24 (92.3)
	Polymicrobial ^e^	2 (7.7)
Infections caused by anaerobic bacteria, n (%)		3 (11.5)
Antimicrobial-resistant infections, n (%) ^f^		3 (11.5)

^a^ Information available for 94 patients. ^b^ Information available for 94 patients. ^c^ Information available for 67 patients. ^d^ Information available for 60 patients. IQR, interquartile range. ^e^ Infections caused by the presence of ≥2 bacteria. ^f^ Antimicrobial resistance of causative organisms was assessed by phenotypic and/or molecular methods. mecA/C and MREJ genes (all detected in *Staphylococcus aureus* organisms) were detected by BJIP but the relevant organisms did not grow in culture in 2/3 of cases.

**Table 3:** Overview of relevant results for synovial fluid samples (N = 17) collected from paediatric patients.

					Synovial Fluid Analysis		Results of:		
Case No.	Sex	Age (year)	Clinical Suspicion	Involved Joint	Appearance	Cell Count/μL (% Neutrophil)	MC	BJIP	Interpretation of Positive Results *
1	F	1	OM	Knee	nd	nd	No growth	*Staphylococcus aureus*	Significant
3	M	1	SA	Hip	Turbid	25,048 (71%)	No growth	*Streptococcus pyogenes*	Significant
7	F	11	SA	Knee	Turbid	2731 (83%)	No growth	None	NA
14	M	17	SA	Wrist	nd	nd	No growth	None	NA
21	F	10	SA	Hip	nd	nd	No growth	*Staphylococcus aureus*	Significant
28	F	6	SA	Ankle	Turbid	279 (55%)	No growth	None	NA
30	M	1 month	OM	Humerus	Turbid	122,030 (-)	No growth	*Haemophilus influenzae*	Significant
54	M	12	SA	Knee	nd	nd	No growth	None	NA
61	M	8	OM	Hip	nd	nd	No growth	*Staphylococcus aureus*	Significant
68	F	2	SA	Knee	Turbid	451 (77%)	No growth	None	NA
71	F	14	SA	Wrist	Turbid	1704 (-)	No growth	None	NA
73	F	2	SA	Ankle	Turbid	38,070 (90%)	No growth	None	NA
77	M	12	SA	Hip	nd	nd	No growth	None	NA
98	M	14	SA	Tibia	Turbid	7424 (98%)	No growth	*Staphylococcus aureus*	Significant
99	M	6	SA	Shoulder	nd	nd	*Streptococcus pyogenes*	*Streptococcus pyogenes*	Significant
102	F	1	SA	Knee	Turbid	2550 (-)	No growth	*Kingella kingae*	Significant
109	F	2	SA	Knee	Turbid	18,642 (-)	No growth	*Kingella kingae*	Significant

F, female; M, male; OM, osteomyelitis; SA, septic arthritis; nd, data not determined; (-), data not reported; * According to clinical suspicion and microbiological findings by MC and/or BJIP; NA, not applicable.

**Table 4:** A comparison between the BJIP and traditional microbiological culture (MC) results for synovial fluid samples from paediatric patients with suspected osteoarticular infections.

Sample Type	Microbial Species	BJIP+ MC +	BJIP+ MC−	BJIP−- MC +	BJIP− MC−	PPA (95% CI)/Adjusted PPA ^a^ (95% CI)	NPA (95% CI)/Adjusted NPA ^b^ (95% CI)	No. Positive Only by BJIP for Samples from Patients Who Were Under Antimicrobial Therapy ^c^:
Synovial fluids (N = 17)	*Staphylococcus aureus*	0	4	0	13	NA/ 100 (39.8–100)	76.5 (50.1–93.2)/ 100 (75.3–100)	4
	*Streptococcus pyogenes*	1	1	0	15	100 (2.5–100)/ 100 (15.8–100)	93.8 (69.8–99.8)/ 100 (78.2–100)	1
	*Haemophilus influenzae*	0	1	0	16	NA/ 100 (2.5–100)	94.1 (71.3–99.9)/ 100 (79.4–100)	1
	*Kingella kingae*	0	2	0	15	NA/ 100 (15.8–100)	88.2 (63.6–98.5)/ 100 (78.2–100)	2
	Total species (n = 9)	1	8	0	59	100 (2.5–100)/ 100 (66.4–100)	88.1 (77.8–94.7)/ 100 (93.4–100)	8

^a^ The “adjusted” PPA was calculated, considering as “true positive” the discrepant results with positive BJIP/negative microbiological culture (BJIP+/MC−), based on the clinical suspicion of POAIs. ^b^ The “adjusted” NPA was calculated considering as “true positive” the discrepant results with positive BJIP/negative microbiological culture (BJIP+/MC−), based on the clinical suspicion of POAIs. ^c^ The 8 species only detected by the BJIP were from 8 synovial fluids samples. PPA, positive percent agreement; NPA, negative percent agreement; CI, confidence interval; NA, not applicable.

**Table 5:** Comparison between the BJIP and traditional microbiological culture (MC, consisting of culture on solid media for pus/biopsies and blood culture for whole blood) results for off-label samples retrospectively collected from paediatric patients with suspected osteoarticular infections.

Off-Label Samples Type	Total	BJIP + MC+	BJIP+ MC−	BJIP− MC +	BJIP− MC −	PPA (95% CI)	NPA (95% CI)	Adjusted PPA (95% CI) ^a^	Adjusted NPA (95% CI) ^b^
Purulent fluids/Biopsies	25	9	6	1	9	90 (55.5–99.8)	60 (32.3–83.7)	93.8 (69.8–99.8)	100 (66.4–100)
Whole blood	68	1	0	0	67	100 (2.5–100)	100 (94.6–100)	100 (2.5–100)	100 (94.6–100)

^a^ The “adjusted” PPA was calculated, considering as “true positive” the discrepant results with positive BJIP/negative microbiological culture (BJIP+ MC−), based on the clinical suspicion of POAIs. ^b^ The “adjusted” NPA was calculated considering as “true positive” the discrepant results with positive BJIP/negative microbiological culture (BJIP+/MC−), based on the clinical suspicion of POAIs. PPA, positive percent agreement; NPA, negative percent agreement; CI, confidence interval.

## Data Availability

All data generated or analysed during this study are included in this published article. Further inquiries can be directed to the corresponding author.
